# Determining Signalling Nodes for Apoptosis by a Genetic High-Throughput Screen

**DOI:** 10.1371/journal.pone.0025023

**Published:** 2011-09-22

**Authors:** Bevan Lin, Derek Huntley, Ghada AbuAli, Sarah R. Langley, George Sindelar, Enrico Petretto, Sarah Butcher, Stefan Grimm

**Affiliations:** 1 Division of Experimental Medicine, Imperial College London, London, United Kingdom; 2 Bioinformatics Support Service, Imperial College London, London, United Kingdom; 3 Medical Research Council-Clinical Sciences Centre, Imperial College London, London, United Kingdom; 4 Department of Epidemiology and Biostatistics, Imperial College London, London, United Kingdom; Stanford University, United States of America

## Abstract

**Background:**

With the ever-increasing information emerging from the various sequencing and gene annotation projects, there is an urgent need to elucidate the cellular functions of the newly discovered genes. The genetically regulated cell suicide of apoptosis is especially suitable for such endeavours as it is governed by a vast number of factors.

**Methodology/Principal Findings:**

We have set up a high-throughput screen in 96-well microtiter plates for genes that induce apoptosis upon their individual transfection into human cells. Upon screening approximately 100,000 cDNA clones we determined 74 genes that initiate this cellular suicide programme. A thorough bioinformatics analysis of these genes revealed that 91% are novel apoptosis regulators. Careful sequence analysis and functional annotation showed that the apoptosis factors exhibit a distinct functional distribution that distinguishes the cell death process from other signalling pathways. While only a minority of classic signal transducers were determined, a substantial number of the genes fall into the transporter- and enzyme-category. The apoptosis factors are distributed throughout all cellular organelles and many signalling circuits, but one distinct signalling pathway connects at least some of the isolated genes. Comparisons with microarray data suggest that several genes are dysregulated in specific types of cancers and degenerative diseases.

**Conclusions/Significance:**

Many unknown genes for cell death were revealed through our screen, supporting the enormous complexity of cell death regulation. Our results will serve as a repository for other researchers working with genomics data related to apoptosis or for those seeking to reveal novel signalling pathways for cell suicide.

## Introduction

Functional, large-scale genetic screens have been undertaken to make sense of the abundance of sequence information from the various genome-wide sequencing projects in order to determine the function of the novel genes. Given the ease of handling and the consistency of their experimental conditions, mammalian cell cultures turned out to be an especially suitable biological system for these screens [1]. Several loss-of-function screens though RNAi-mediated gene reduction have been reported [2], [3]. However, such screens only reveal a subset of pathway components, either because genes often exert redundant functions or the contributions of the respective factors are too small to be detectable. Hence, gain-of-function screens for dominant gene activities can likewise reveal important functional data since the genes can activate signalling pathways in which their respective proteins are rate-limiting.

Apoptosis is the genetically regulated cell suicide programme. It is especially amenable to screens since selection schemes cannot be implemented for dying cells. Moreover, the life/death decision of the cell is likely to be influenced by many cellular processes with numerous, possibly so far unknown, positive and negative regulators. Also, many mediators of apoptosis signals often have the dominant activity to induce apoptosis when ectopically expressed as they exert their effect through protein-protein interactions, which are induced when they are upregulated.

We have established a systematic screen for dominant gene functions [4], [5] named RISCI (robotic single cDNA investigation) [1]. For this, we grow up individual expression plasmids in bacteria in a 96-well plate format. The plasmid DNAs are isolated using a special protocol that yields DNA that is virtually devoid of endotoxins and allows for efficient transfections of cells *in vitro* [6]. Since we are testing the activities of individual genes in separate populations of transfected cells, we obtain favourable signal-to-noise ratios and a high sensitivity in our functional read-out. Each of these apoptosis genes, when overexpressed, initiates a signalling pathway that eventually ends in the active dismantling of the cell.

In a previous study we reported 11 apoptosis-inducing genes from a manual screen [5], which produced an especially strong apoptosis phenotype. Some isolates were already known cell death inducers and served as positive controls. The specificity of this screen was emphasized by the finding that only particular genes from gene families were active [7], [8], [9]. Also, genes that could potentially activate general stress pathways such as ER stress did not score positive, nor did dominant-negative genes or constitutively active oncogenes [5]. Importantly, the screen permits the detection genes that have not been implicated in the apoptosis response previously. Using additional information from extensive literature scans and careful sequence analysis we have chosen some genes for further investigation [5], [7], [9], [10], [11], [12], [13], [14]. None of them were known to induce cell death at the time of their discovery in our screen and all of them have been shown to mediate upstream signal for cell death. We have since constructed custom-made robots for the screen [15], [16]. These machines are highly specialised and permit the screening of large numbers of genes. Here we reveal the full potential of this screen and present a comprehensive account of our screening activities with the robots in combination with a thorough bioinformatics analysis of the isolated genes.

## Results

### Isolating of apoptosis inducing genes in a high throughput screen

**Figure 1:**
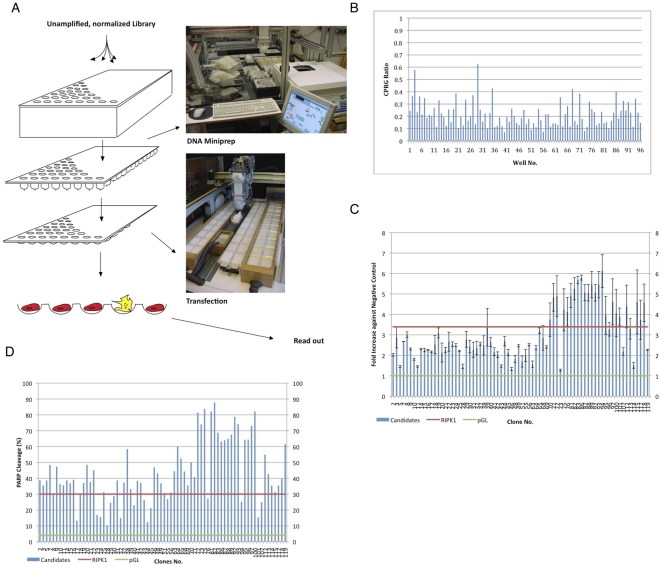
Isolation of apoptosis genes with a robotic screen. **A.** Schematic illustration of the screen for apoptosis genes. Individual bacteria clones are grown up in 96-deep well plates and their plasmid DNA is harvested using a custom-made DNA isolation robot. A second robotic system introduces the plasmids into mammalian cells and the cellular effect is detected by a read-out that can be assessed by a plate reader. **B.** Example of a β-galactosidase assay result that was used to determine cell death. The ratios of the A_590_ of CPRG before and after lysis of the cells were determined 40 hours after transfection and are plotted against the well numbers. Well #3 contained the positive control. **C.** Validation of the cell death genes using an ELISA specific for apoptosis. Ultra-pure plasmid DNA of the various plasmids were prepared as described in [12] and transfected into HEK 293T cells using jetPEI (PolyPlus) transfection kit. The assay for apoptosis was performed 48 hours post transfection with Cell Death Detection ELISA (Roche) detecting nucleosomes in cytoplasmic fractions as described by manufacture's protocol. Absorbance is measured at 405 nm and the apoptosis enrichment factor calculated as the signal of individual clones against the negative control, GFP. The red and green lines indicate apoptosis by the positive and negative controls, RIPK1 and GFP, respectively. **D.** Validation of the cell death genes using a Western blot for PARP cleavage. HEK 293T cells were transfected as under C. and 48 hours later cells were harvested and lysed in RIPA buffer. Proteins were separated on a 12% SDS-PAGE gel, blotted onto a membrane, and probed with a PARP antibody. Using the programme ImageJ the percentage of cleaved PARP was determined for each gene.

We screened a normalized mouse kidney cDNA library for apoptosis inducers using our experimental screen set-up (Fig. 1A) in which two steps, the DNA isolation and the transfection, are performed by custom-made robots. After the DNA isolation robot had purified the plasmid DNAs, the transfection robot introduced them individually into cells. Per day this high-throughput screen achieved 1,536 separate DNA-isolations, -transfections and activity assays for apoptosis. We used HEK293T cells (human embryonic kidney cells) as we found them to be easily transfectable and consistently sensitive to apoptosis inducers. The activity of a co-transfected and internally expressed β-galactosidase was measured by the addition of its substrate CPRG (chlorophenolred-ß-D-galactopyranoside) into the medium. The transient co-transfection of the reporter plasmid compensates differences in the transfection efficiency between wells. This assay probes the permeability of the plasma membrane for small molecular weight reagents upon cell death induction. This is then normalised to the transfection efficiency, which is revealed upon lysis of the cells. Figure 1B shows a typical example of a screen result with the ratios of the CPRG measurements before and after addition of the detergent plotted against the numbers of wells in a 96-well plate. Hits were identified when the ratio was higher than 0.5, which corresponds to a 50% conversion of the CPRG substrate. We have screened 100,000 expression constructs of a normalized cDNA library. As the screen is based on scoring cell death by the permeabilisation of the plasma membrane, which can also be observed in cell death modes other than apoptosis such as necrosis, we validated the isolated genes with an enzyme-linked immunosorbent assay (ELISA) that detects the DNA fragments generated during apoptosis. 75 (87%) of the isolated genes achieved ratios higher than the green fluoroscent protein (GFP) negative control and hence were regarded as positive in this assay (Figure 1C). We also use the cleavage of endogenous PARP (Figure S1), a known substrate of caspase-3 as an indicator of apoptosis activation [17]. All clones except one showed caspase-3 activity upon ectopic expression with a general trend in the efficiency similar to the ELISA assay (Figure 1D). Only those genes were subjected to a thorough bioinformatics analysis.

### Functional annotation of the isolated genes

**Figure 2:**
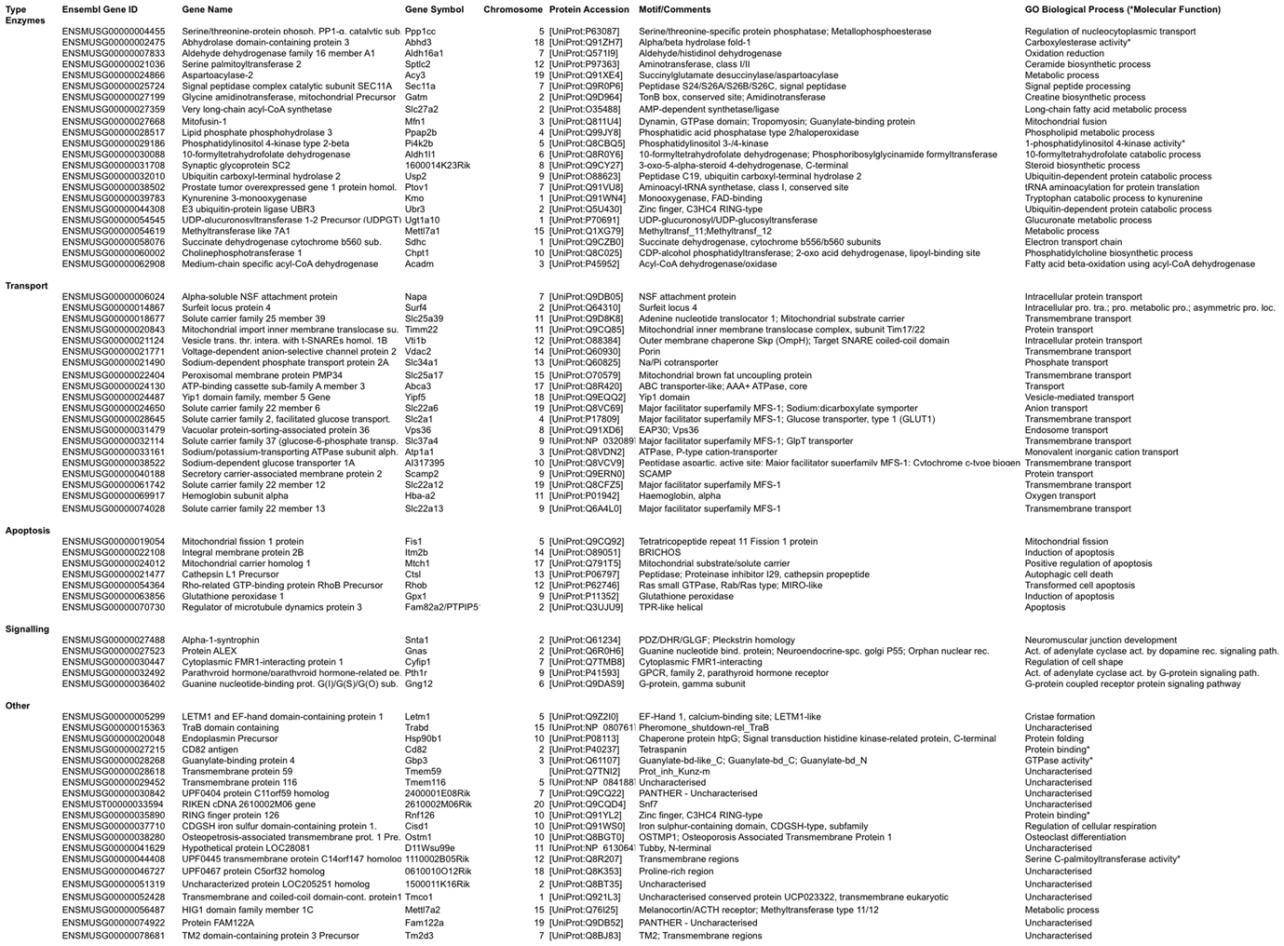
Collection of apoptosis genes isolated by the screen. The identities of the pro-apoptotic genes are listed, allocated to the most prominent functional categories, enzymes, transporters, apoptosis and signalling factors, together with their ENSEMBL identifiers, gene names, protein accession numbers, salient InterPro motifs and biological processes from the GO data base. In the last column “Uncharacterised” on its own means that there are no defined motifs in the respective sequence. “Uncharacterised-PANTHER” entries refers to genes that contain a domain/motif that has been identified by Panther but the function is unknown.

Figure 2 I records the genes grouped into different functional categories starting with enzymes and transporters, the two most prominently represented gene functions, totalling 43 genes, followed by known apoptosis genes, generic signalling factors, and genes without a unambiguous functional association (“Other”). We also list their Ensembl number, gene name and symbol, chromosome- and protein accession-number, motifs as well as biological processes/functions as determined with the database Biomart. 7 genes (9%) were found to have a known role in apoptosis regulation, which therefore serve as positive controls: Among them is the Mitochondrial carrier homolog 1 (*Mtch1*), also known as *PSAP*, a transporter in mitochondria that interacts with and could mediate apoptosis by presenilin-1 in Alzheimer's disease [18]. Three enzymes were among the known apoptosis genes: cathepsin L, glutathione peroxidase-1, and the GTPase *Rhob*, all of which have been shown to mediate apoptosis signals [19], [20], [21]. Likewise, *Itm2b*, *Fam82a2/PTPIP51*, and the mitochondrial fission factor *Fis1* are known as apoptosis regulators [22], [23], [24]. Itm2b has been described as a novel BH3-only protein [25] but little else is know about it. The protein tyrosine phospatase interacting protein 51 (PTPIP51), also known as Fam82a2, is a substrate for PTP1B/TCPTP protein tyrosine phosphatases and, based on its many protein interactions, seems to be positioned at a crucial nexus for a variety of signalling pathways, including apoptosis [26]. Fission factors such as Fis1 are likewise well-known apoptosis mediators [27].

**Figure 3:**
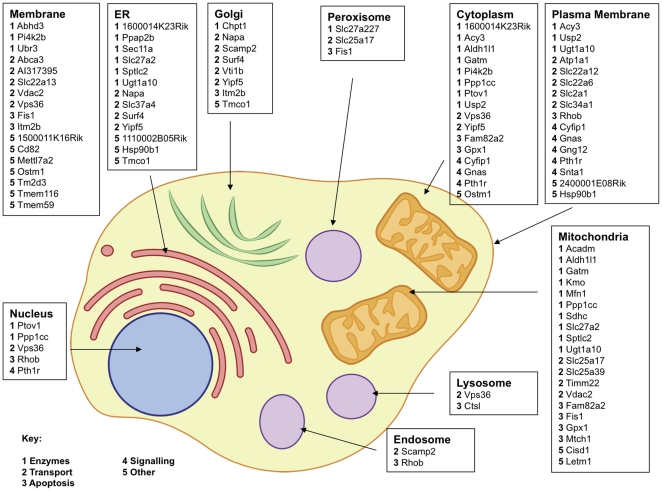
Predicted subcellular distribution of the apoptosis genes. The localisation of the gene products was determined by the GO (The Gene Ontology Consortium [28]) cellular component ontology using BioMart [29]) and allocated to the respective insert with other genes predicted to be localised to the same organelle. Multiple listings were allowed. The functional class of each gene is indicated by a number and refers to figure 2. Key: 1 Enzymes, 2 Transport, 3 Apoptosis, 4 Signalling, 5 Other.

We then determined the subcellular localisation of the apoptosis factors that we found in the screen from the GO (The Gene Ontology Consortium [28]) cellular component ontology using BioMart [29] (Figure 3) and also performed an enrichment analysis with DAVID (Table S1). Various cellular organelles are involved in apoptosis regulation and it has been proposed that each of them contains sensors for cellular damage that can signal apoptosis [30], [31]. This is supported by our findings as we detected apoptosis proteins in all subcellular compartments. In keeping with the prominent role of mitochondria for the induction of apoptosis, this organelle is predicted to harbour the largest number of all isolates from the screen and of the known apoptosis factors. The accumulation of apoptosis factors in mitochondria is likely to reflect the complexity of apoptosis regulation in this organelle. The number of novel apoptosis regulators localized to this organelle (16 out of 20) indicates that the complexity is even greater than so far recognized and suggests that the mitochondrion is still a rich source of so far unknown apoptosis circuits.

The endoplasmic reticulum (ER) is, besides mitochondria, another well-known membrane-enclosed compartment governing cell death. Its role for cell death regulation is also underscored by our results as the organelle with the second largest number of isolates from the screen. One of the crucial steps by which the ER contributes to apoptosis is the release of Ca^2+^. Consistent with this, this organelle comprises a number of transporters from the screen that could, directly or indirectly, facilitate this process. The Golgi apparatus has only recently been implicated in apoptosis regulation [32]. During apoptosis this organelle undergoes dramatic structural changes and eventually disintegrates, most likely as a result of the cleavage of Golgi proteins. Interestingly, one of our positive controls for apoptosis, ITM2b is localised to this organelle. This gene is also known as *Bri* and is associated with British familiar dementia [33], possibly by interfering with amyloid-β (Aβ) processing [33]. The peroxisome is mostly known for its activity to repress apoptosis. This stems form the activation of peroxisome proliferator receptors, nuclear transcription factors that mediate an accumulation of peroxisomes, but these organelles have also other, independent functions [34]. Our data indicate a pro-apoptotic activity of the peroxisome. Interestingly, the endosome as well as the lysosome, which develops from an early endosome, exhibit apoptosis factors. The lysosome is, besides mitochondria and the ER, another organelle that is thought to release specific pro-apoptotic factors (such as cathepsin L, Figure 2) [19]. Also, the endosome has recently emerged as impacting on apoptosis. A gene from *H. pylori* causes Bax accumulation at endosomes and the close alignment of endosomes with mitochondria before Bax is retrieved at mitochondria [35]. The scarcity of nuclear factors from the screen (7%), relative to the complexity of this organelle, is noteworthy and possibly reflects that, while nuclear signals can cause apoptosis, the nucleus is a compartment that is mostly not required for cell death induction [36]. Interestingly, of all genuine signalling molecules from the screen only Pth1r can be found in the nucleus, all others are confined to the plasma membrane and the cytosol.

**Figure 4:**
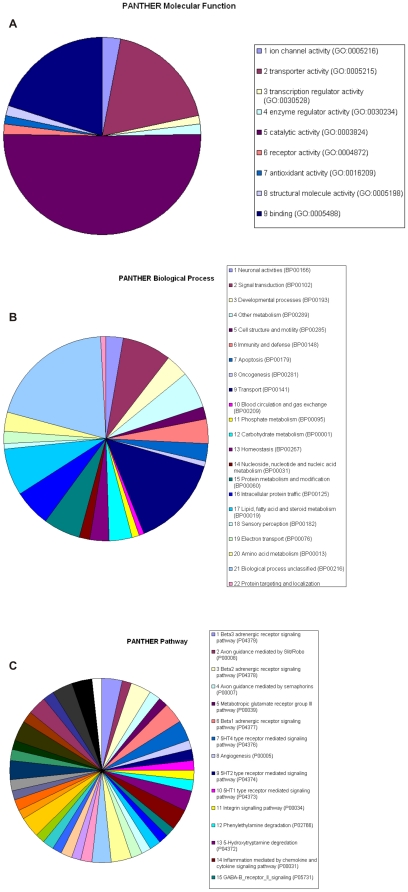
Allocation of the apoptosis genes to functions, biological processes and signalling pathways. **A.** The pie chart depicts the allocation of the apoptosis genes to molecular functions according to the PANTHER database. **B.** Grouping of the main biological processes governed by the genes from the screen. **C.** Assembly of the known signalling pathways that are regulated by the genes from the screen.

According to the information in the PANTHER database the pie chart in Figure 4a splits the functions into further functional categories compared to Figure 2, which again illustrates the abundance of transporters and enzymes among the isolated genes. The third most prominent functional classification was the binding category, which supports our view that much of the signalling for apoptosis is mediated by protein-protein interactions. In agreement with the underrepresentation of nuclear factors from the screen, only few isolates fall under the category of transcription regulatory activity.

Clustering the genes for biological processes revealed a prominent role for transporters and the fact that a substantial number of genes could not be integrated into a known biological process and hence are labelled as “unclassified” (Figure 4b). When we investigated the known signalling pathways represented by the genes we found that no clustering occurred when using the PANTHER database (Figure 4c). This suggested that the isolated factors define signalling pathways that are separate from the ones they engage in healthy cells. In fact, it is well known that apoptosis factors often have completely different functions in non-apoptotic cells [37]. This finding indicates that during apoptosis the components of the apoptosis signalling pathways are recruited from a diverse set of signalling circuits that are unrelated to cell death.

**Figure 5:**
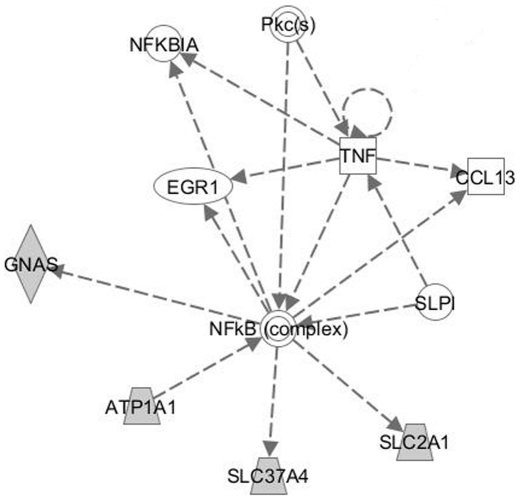
Signalling pathway identifications. Signalling pathway links were generated through the use of Ingenuity Pathways Analysis (Ingenuity® Systems, www.ingenuity.com).

In an effort to connect the isolated genes and define signalling pathways, we used the Ingenuity Pathways Analysis (Ingenuity® Systems, www.ingenuity.com) and found that several isolates are linked through the TNF/NF-κB signalling pathway (Figure 5). This protein complex regulates both pro- and anti-apoptotic genes and is activated under cell stress conditions. Three target genes (*Gnas, Slc2a1, Slc37a4*) were found and *Atp1a1* has been reported to signal via the inositol 1,4,5-trisphosphate receptor to activate NF-κB [38].

### Comparison with microarray data

**Figure 6:**
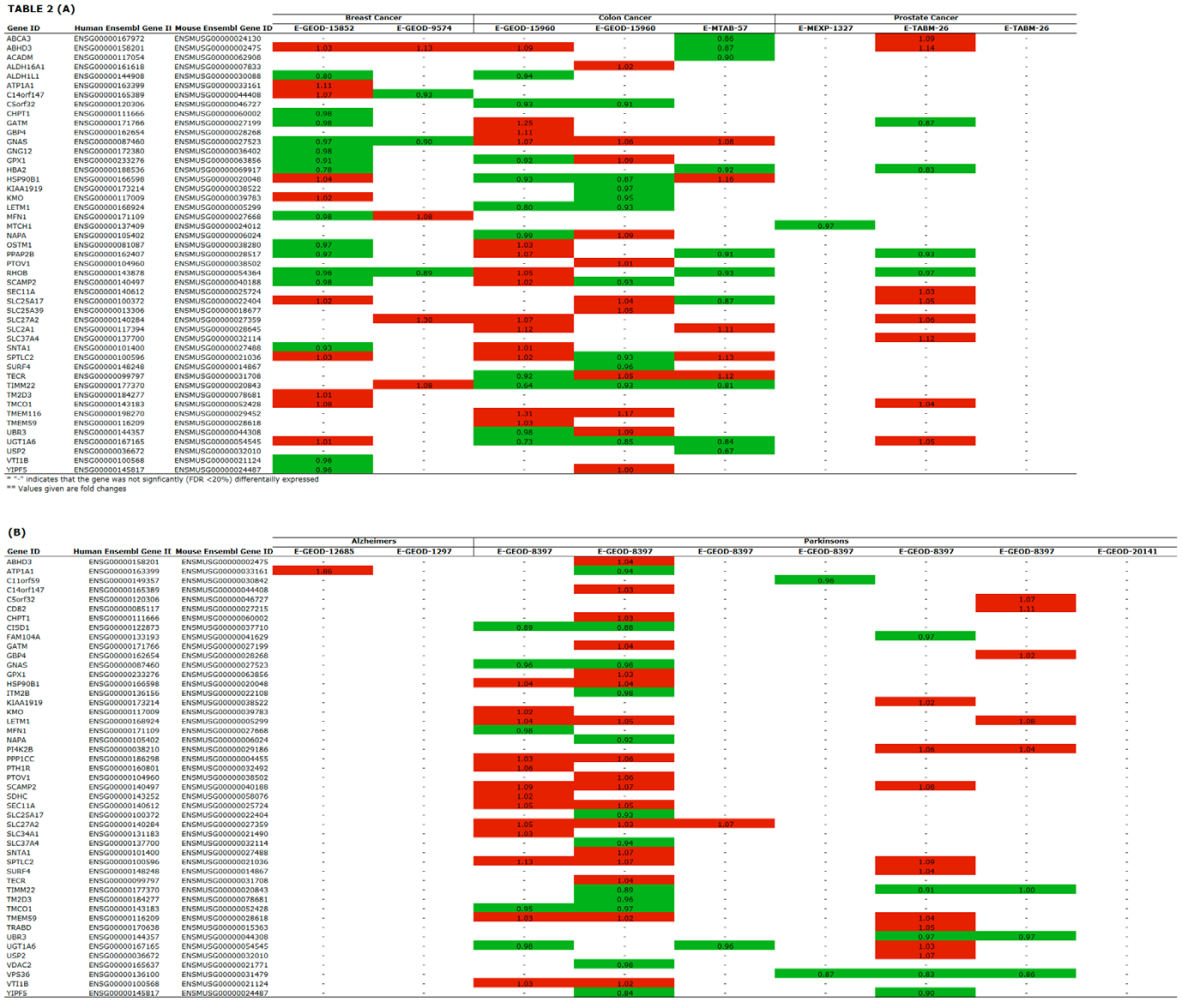
Differential expression of 71 human orthologues of the 74 mouse genes in publically available gene expression datasets. Not all 71 of the human orthologues were found to be differentially expressed within the publically available datasets at a 20% FDR rate. The numbers are fold changes; red represents an over-expression of the disease cell type and green represents an under-expression of the disease phenotypes. Both human and mouse Ensembl IDs are given as well as the dataset ArrayExpress ID. A.) Breast, colon and prostate cancer disease types. B.) Alzheimer's and Parkinson's disease types.

In figure 6A we compared our isolates with differentially expressed genes by microarray analysis on breast cancer (two studies), colon carcinoma (three studies), and prostate cancer (three studies). We recorded genes that were found both up- and downregulated (see Methods). Apoptosis genes from the screen whose expression was reduced would indicate an impairment of pro-apoptotic signalling in the tumour cells. Those genes that were upregulated are likewise of interest as they could indicate that these cancer cells continue to grow, despite experiencing pro-apoptotic stress. The oncogene *myc*, for example, exerts both a proliferative effect as well as a pro-apoptotic signal [39]. In those tumour cells anti-apoptotic signalling molecules such as the Bcl2 family members are potential targets for treatment. While a considerable heterogeneity in the transcriptional changes of the genes can be observed, some genes show consistent alterations. The hemoglobin gene (*Hba-a2*), for example, was found to be downregulated in all three cancers. UDP-glucuronosyltransferase 1–2 Precursor *(UDPGT)/Ugt1a10*) and the mitochondrial import inner membrane translocase subunit (*Tim22*) gene were reduced in all three colon cancers; as was the *ALEX* (*Gnas*) gene in both breast cancer studies. On the other hand, the same gene was consistently upregulated in colon cancer. Interestingly, a recent report confirmed that *Gnas* is mutated in several types of cancers [40]. Other upregulated genes are solute carrier family 2, facilitated glucose transporter member 1 (*Slc2a1*) and transmembrane protein 116 (*Tmem116*). Abhydrolase domain-containing protein 3 (Abhd3) is upregulated in breast cancer tumours.

We also looked at microarray studies performed on tissues from degenerative diseases such as Alzheimer's (2 studies) and Parkinson's Disease (7 studies). We reasoned that genes that are downregulated in these diseases could indicate a response by the cells to reduce their sensitivity to cell death signals. Hence, we also present those genes whose transcription level declined. Figure 6B shows that for the two available microarray studies on Alzheimer's only the sodium/potassium-transporting ATPase subunit alpha-1 precursor (*Atp1a1*) gene was detected as changed (P = 0.009). Except for one gene the transcriptional changes in the Parkinson's studies were much more consistent, i.e. the genes are either up- or downregulated. Many genes contribute only incrementally to the observed phenotype [41] and the combination of the functional screen with microarray data could identify this group of genes.

## Discussion

Our screen for apoptosis genes has revealed a host of novel factors that have previously not been implicated in cell death regulation. Since each isolate is capable to initiate a downstream signalling pathway that eventually converges on the activation of the pro-apoptotic caspase proteases, the complexity and the vast number of cellular nodes that can regulate apoptosis becomes apparent. While we have isolated a number of positive controls, most of the genes that are known to regulate apoptosis were so far not discovered by the screen. Hence, our screen can be regarded as a first step to cover the whole genome for apoptosis genes, which will yield a inventory of its signalling nodules and allow mapping the “functome” [42] of apoptosis. The positive controls of known apoptosis genes represent less than 10% of the genes determined in this study (Figure 2) with many apoptosis genes such as caspases still missing. How many genes in the genome are involved in apoptosis? If we take as reference a compilation of known apoptosis genes [43], which lists 110 genes in *H. sapiens*, and extrapolate our data on known apoptosis inducers to the complete genome, assuming that the percentage of so far undiscovered apoptosis inducers correlates with the percentage of positive controls from the screen, this would result in a total of more than 1,000 genes involved in apoptosis. This supports the hypothesis that many additional genes exist that impact on apoptosis.

The smallest group (7% of all isolates) in figure 2 subsumes those genes that can principally be regarded as signalling factors. The scarcity of such genes indicates that apoptosis signalling is performed via different routes compared with most other signalling pathways. The largest gene group from the screen comprises enzymes (30%). All enzyme classes were represented among the isolates except lyases and isomerases. On the other hand, the occurrence of kinases, the classical signalling mediators for many other processes such as the cell cycle was only minor suggesting that many signalling pathways in apoptosis contain other or additional components. Also, the high number of transporters (27%) in figure 2 is intriguing. When we clustered the genes according to their main biological process, we again discovered a prominent role for transporters (Figure 4b). Apoptosis can be caused when boundaries between organelles break down. This is best known for the disruption of the outer mitochondrial membrane and the release of apoptotic factors such as cytochrome c and AIF [44]. Indeed, mitochondria have the highest number of transporter proteins of all organelles (Figure 3), again highlighting that the process of the permeabilisation of mitochondrial membranes is crucial for apoptosis. Our results indicate that additional proteins are involved in this process. This is in agreement with the fact that the identity of the proteins that facilitate the loss of the integrity of mitochondrial membranes during apoptosis and constitute the “permeability transition pore” (PT-pore), is still unresolved [45].

In the apoptosis field the general distinction between extrinsic and intrinsic pathways is often made [46] with the extrinsic pathway operating through membrane receptors and the intrinsic through mitochondria activation. Our functional annotation indicates that only a minority of genes from the screen code for receptors (Figure 3) and hence the pathways to report cell stress to mitochondria are much more divers.

If half of the chemicals in the Sigma catalogue cause apoptosis at high enough concentrations [47], can it be a good idea to screen for dominant apoptosis-inducing genes? Might they not only unspecifically damage the cell and hence reveal little about apoptosis signalling? Our studies on some of the genes from the screen indicated that genes closely related to those apoptosis inducers do not cause apoptosis [9], [12]. Of note, we have not isolated many proteins localised to the ER. An accumulation of proteins at this organelle can lead to what is subsumed under “ER stress”, and can, if prolonged, cause apoptosis. Control experiments with genes whose proteins are directed through the ER were negative for apoptosis as were numerous oncogenes and dominant-negative gene variants [5]. All genes from our screen induce a downstream apoptosis signalling pathway that ultimately results in the activation of caspases since both read-outs, the ELISA (Fig. 1C) which depends on the degradation of the DNA, and the PARP cleavage (Fig. 1D) are induced by these proteases [48], [49]. We have also employed in the experimental setting of the screen that the apoptosis response is evolutionary conserved by using mouse genes in human cells. Collectively, these aspects indicate that, in contrast to unspecific cell stress exerted by many small molecular weight compounds [47], the genes from the screen cause specific signals in the cell that define pro-apoptotic signalling circuits. Whether the isolated genes also mediate upstream signals for apoptosis, i.e. whether exogenous signals talk to the endogenous proteins of our isolates, can only be answered on a case-by-case basis. With those genes that we further investigated, we found that upstream signals for apoptosis were indeed inhibited when the genes were inactivated [5], [7], [9], [10], [50]. Our results with microarrays (Figure 6) indicate that the genes from the screen are potentially also involved in disease scenarios. Given that apoptosis is an active response by the cells, similar to other differentiation programmes, we speculate that eventually all of the isolates can be integrated into a physiological or pathological context of apoptosis induction.

One of the most important applications of the apoptosis genes presented in this work will be the annotation of genes that are differentially expressed by microarray analysis, which we exemplified in figure 6. Our functional data can in particular contribute to the massive sequencing efforts to distinguish those mutations underlying tumourigenesis in the “cancer genome” from those that are mere “passengers” [51]. As it is the upregulation of the genes that activates them for apoptosis, we would expect that it complements microarray data, which record such transcriptional changes. This combination will add an important functional aspect to the correlative data produced by microarrays. A case in point is the determination of hemoglobin as an apoptosis inducer in our screen (Figure 2) and in microarray analysis [52]. While surprising, hemoglobin has been found in later studies to be upregulated in various cell types and responsible for apoptosis [53], [54].

After our first reports on the apoptosis screen [4], [5], [55] and its technology [15], [16], [56] similar screens for dominant apoptosis inducers were published by other groups [57]. One approach isolated a number of genes that were described as potential tumour suppressor genes [58]. Other researchers screened and identified three genes out of 938 hypothetical genes as apoptosis inducers using standard transfections in combination with subcellular imaging, western blotting, and DNA fragmentation ELISA [59]. The most comprehensive effort so far to screen for cell death-inducing genes was performed by testing pools of genes each comprising 96 cDNAs from a T-cell library. This screen produced, besides factors that also induce alternative phenotypes of cell death, 64 genes for apoptosis induction [60]. Interestingly, only three genes (*MTCH1*, *GNAS*, *SDHC*) overlap with our gene collection possibly indicating differences between the libraries used. Other technical approaches have been employed to screen for apoptosis mediators. Reverse transfection cell array technology (45) arrays the transfection mixes, each containing an individual gene of interest, onto glass slides, which are then overlaid with cells. With this approach a collection of 382 human sequence-verified, full-length open reading frames were screened for proapoptotic genes and seven genes were identified. A similar cell array screen was also performed with 1,959 mammalian open reading frames from the Mammalian Genome Collection with 10 proapoptotic genes eventually being verified (46).

In summary, we believe that this study demonstrates that testing gene activities by individual transfections holds great promise to the definition of the functome of apoptosis and can have applications in other functional read-outs as well.

## Materials and Methods

### Screening

The various steps of the screening procedure were performed as described using our custom-made robots [5], [15], [55]. The DNA isolation robot performed our special protocol of DNA isolation that yields exceptionally pure DNA that can be transfected efficiently into cells and does not generate background cell death – as published [16]. Some of the description is available online as part of our publication of the DNA isolation robot [16]. Screening was done in duplicates. Only these genes that are active in both assays were regarded as positive. An expression vector for luciferase was used as negative control. The integrity of the cell membrane was probed by adding the β-galactosidase substrate CPRG, which can enter dying cells and is converted into a coloured product by the enzyme. The cells are then lysed by TritonX-100 in order to normalize for the transfection efficiency [15]. For the read-out we made use of the FLUOROstar microplate reader from BMG Labtech and the data were processed by the Windows-based OPTIMA software on a DELL Dimension 3100 computer.

### Apoptosis detection

The Cell Death Detection ELISA (Roche), which detects nucleosomes in cytoplasmic fractions, was used as described in manufacture's protocol. For the PARP Western blotting HEK 293T cells were transfected using jetPEI transfection kit (Polyplus-transfection, France) according to manufacturer's protocol. 48 hours post transfection the cells were lysed with RIPA buffer. Western blot was performed with a PARP antibody (Cell Signalling #9542) and developed with ECL (Pierce) and Amersham Hyperfilm (GE Healthcare). Analysis and quantification of the proteins was performed using ImageJ.

### Bioinformatics analysis

The associated Ensembl [61] gene IDs for the mouse cDNAs were identified by BLAST [62] using blastx to search the Ensembl peptide database (NCBIM37.58). Using the identified Ensembl gene IDs the gene names, UniProt accessions, InterProScan [63] motif/domain information and GO (The Gene Ontology Consortium [28]) biological process, molecular function and cellular location information were all extracted from the Ensembl Genes 58 Mus musculus genes (NCBIM37) dataset in BioMart [29]. The PANTHER [64], [65] molecular function and pathway information was obtained from the PANTHER web site (version 7.0) using the Ensembl gene IDs.

### Differential gene expression studies

Publically available gene expression datasets for breast, colon and prostate cancer as well as Parkinson's disease and Alzheimer's disease were downloaded in a processed format. The datasets were either Affymetrix GeneChip Human Genome HG-U133A, Affymetrix GeneChip Human Genome HG-U133B or Affymetrix GeneChip Human Genome U133 Plus 2.0 gene chips. The gene expression datasets were analyzed in R using the *affy* [66] and *preprocessCore* [67] Bioconductor [68] packages. Each gene expression dataset was log transformed and normalized by quantiles across the array. Probes were then filtered out by the Affymetrix detection calls, whereby probes that were “present” or “marginal” were kept and those that were “absent” were removed. The probes that were the least varying, the bottom 10% as determined by the coefficient of variation, were removed as well. For datasets containing multiple tissues, each analysis was performed within tissues. Differentially expressed genes were identified using the Significance Analysis of Microarrays (SAM), found in the *siggenes* [69] package in Bioconductor. SAM utilizes a modulated t-test to determine differential expression and permutation tests were used to correct for multiple testing. Differentially expressed genes were identified at a False Discovery Rate of <20% for comparison with the mouse apoptosis genes. 71 out of 74 mouse apoptosis genes had human orthologues; figure 6 shows which of those genes were differentially expressed for a given dataset. For each gene present, the fold change was calculated whereby the values >1 represent over-expression in the disease state.

## Supporting Information

Figure S1 **Accumulation of cleaved PARP as an assay for caspase-3 activity.** A Western blot of extracts from cells transfected with a selection of clones with a representative range of different PARP cleavage activities together with negative (pGL) and positive (RIPK1) controls is shown. Equal loading was verified with β-actin (bottom). Band intensities and conversion ratios were calculated with ImageJ (top).
(TIF)

Table S1 **Enrichment of GO and pathway (KEGG and Panther) terms within the 74 mouse genes.** The enrichment analysis was performed in DAVID* with the search results restricted to gene ontology (GO) terms and KEGG and Panther Pathways. The terms highlighted in blue are significant at a 10% FDR level, as determined by the Benjamini method. The GO/Pathway identifiers are listed along with the genes involved and the relevant statistics.
(XLS)
