# Supplementary figures and images for: Determining Signalling Nodes for Apoptosis by a Genetic High-Throughput Screen
Source: PLoS One. 2011 Sep 22;6(9):e25023. doi: 10.1371/journal.pone.0025023 (PMC3178610; doi:10.1371/journal.pone.0025023)

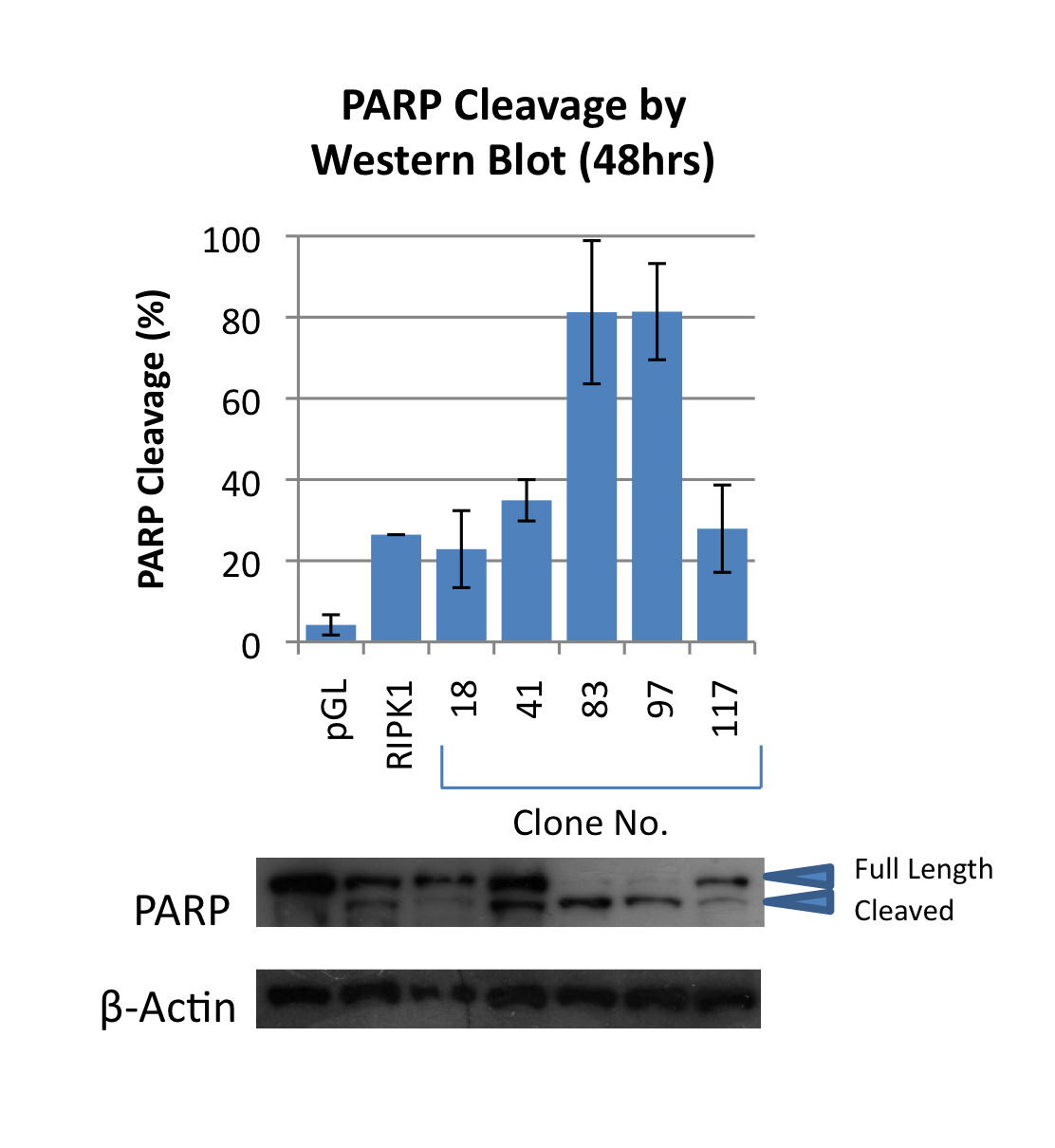

Supplement: Figure S1 — Accumulation of cleaved PARP as an assay for caspase-3 activity. A Western blot of extracts from cells transfected with a selection of clones with a representative range of different PARP cleavage activities together with negative (pGL) and positive (RIPK1) controls is shown. Equal loading was verified with β-actin (bottom). Band intensities and conversion ratios were calculated with ImageJ (top). (TIF) [file pone.0025023.s001.tif]
